# Source apportionment and non-carcinogenic health risk assessment of selected heavy metals in the Turag river area of Gazipur District, Bangladesh

**DOI:** 10.1371/journal.pone.0362351

**Published:** 2026-11-06

**Authors:** Juned Hussain Chowdhury, Md Jamil Hossain, Ryadul Alam, Ifteker Uddin Sakib, Wahida Ahmed, Naimur Rahman Sarker, Sangita Chakroborty, Md Mushfiqur Rahman Porosh, Md Shakhaoat Hossain, Badhan Saha, Mohammad Moniruzzaman, Ayesha Ahmed

**Affiliations:** 1 Department of Public Health and Informatics, Jahangirnagar University, Savar, Dhaka, Bangladesh; 2 Health and Environmental Epidemiology Laboratory (HEEL), Department of Public Health and Informatics, Jahangirnagar University, Savar, Dhaka, Bangladesh; 3 Bangladesh Council of Scientific and Industrial Research, Dhaka, Bangladesh; 4 Climate Change, Air Quality, and Health Research (C2AHR) Unit, Department of Public Health and Informatics, Jahangirnagar University, Savar, Dhaka, Bangladesh; National Autonomous University of Mexico Institute of Geophysics: Universidad Nacional Autonoma de Mexico Instituto de Geofisica, MEXICO

## Abstract

**Introduction:**

Rapid industrialization in Bangladesh, especially in Gazipur District, has raised concern over heavy metal contamination of soil and water and the associated health risks to nearby residents. This study compared the concentration, spatial distribution, probable sources, and non-carcinogenic health risk of five heavy metals (Hg, Ni, Mn, Zn, and Co) in soil and water in the Turag River areas in Gazipur District.

**Methods:**

Soil and water samples were collected from 18 sites in the Turag River (including both industrial and non-industrial zones) and analyzed for five heavy metal concentrations (Hg, Ni, Mn, Zn, and Co) using inductively coupled plasma-mass spectrometry (ICP-MS). Statistical analyses included principal component analysis (PCA), hierarchical cluster analysis (HCA), and Pearson correlation analysis, while non-carcinogenic human health risks were evaluated using the USEPA-based chronic daily intake (CDI), hazard quotient (HQ), and hazard index (HI) models for both adults and children.

**Results:**

Mean soil metal concentrations followed the order Mn > Zn > Ni > Co > Hg in both zones, with markedly higher and more spatially concentrated Ni and Zn levels in the industrial zone near the Turag River areas. Spatial mapping, PCA, HCA, and correlation analyses consistently suggested a common anthropogenic influence on soil and water contamination in the industrial zone, with the strongest spatial concentrations observed near its northern boundary, compared with patterns consistent with multiple diffuse influences in the non-industrial zone. The modeled soil CDI values were generally higher for children than adults. Soil-based screening estimates indicated potential non-carcinogenic concern for children, including an Ni HQ of 1.66 in the industrial zone and cumulative hazard index values above the screening benchmark of 1 for children in both the industrial (HI = 2.32) and non-industrial (HI = 1.31) zones, while all modeled HQ and HI values for water remained below the screening benchmark of 1.

**Conclusion:**

These soil-based screening estimates suggest potential non-carcinogenic concern for children, particularly regarding Ni in the industrial zone, but are based on the < 0.5 mm fraction of soil collected at 15–20 cm depth and may not represent actual exposure to ground-surface soil or airborne particles. The study highlighted the need for targeted control of potential industrial sources and child-focused environmental health monitoring in industrializing peri-urban districts of Bangladesh.

## 1. Introduction

Heavy metal contamination is one of the most difficult environmental hazards of the twenty-first century, considering prevalent urbanization, industrialization, and unsustainable farming practices [1,2]. Unlike organic pollutants, heavy metals are non-biodegradable and can persist in the environment, accumulating through biogeochemical cycles and food chains [3]. They can be released into the environment both naturally and as a result of human activities [4]. The concentration of heavy metals in the environment has greatly increased due to rapid industrialization and urbanization, mining, metal processing, fossil fuel combustion, electroplating, and extensive use of chemical fertilizers and pesticides, which have resulted in widespread contamination of terrestrial and aquatic environments [5].

Wastewater discharge is a major contributor to heavy metal contamination. Due to their non-biodegradable nature, these metals accumulate in sediments and aquatic organisms, where they can bioaccumulate in the food chain [6]. As a result, heavy metal pollution has become a major concern for aquatic ecosystems and can adversely affect fish and other edible aquatic organisms. Human exposure occurs primarily through the consumption of contaminated aquatic products, although heavy metals may also accumulate in food crops and other food products [7–10].

Developing nations undergoing rapid industrialization and urbanization face disproportionate challenges from heavy metal contamination. Global assessments have reported lower heavy metal concentrations in developed countries across Europe and North America compared to developing regions in South America, Asia, and Africa, with mining, manufacturing, and inadequate industrial waste controls identified as major pollution drivers [11,12]. As a South Asian country, Bangladesh has made a tremendous leap in industrial development over the last two decades, driven by its textile, pharmaceutical, chemical, and metal processing industries [13]. This rapid expansion has not been matched by corresponding investments in environmental infrastructure or pollution control, resulting in extensive contamination of surface waters and soils [14]. Industrial effluents, poorly managed or untreated, are frequently released into rivers; urban waste and agricultural runoff also contribute to pollution. Previous studies reported that major river systems associated with industrial areas, including the Buriganga and Turag, contain very high levels of heavy metals [14–16], exceeding drinking-water and irrigation standards, with potential implications for aquatic ecosystems and downstream populations [16,17].

Gazipur District, located immediately north of Dhaka, has experienced rapid industrial expansion in recent years [18]. The Department of Environment, Bangladesh, has identified more than 450 polluting industries across Dhaka Division, with a high concentration of textile, dyeing, and pharmaceutical industries in Gazipur [14,19]. Textile dyeing, pharmaceutical, and chemical manufacturing all generate heavy-metal-bearing wastewater, and the literature consistently identifies mercury (Hg), nickel (Ni), zinc (Zn), manganese (Mn), and sometimes cobalt (Co) as recurring primary or secondary contaminants across those streams [20,21]. Among these heavy metals, Hg is widely used in products such as fluorescent lamps, batteries, and electronic devices, and improper disposal of these materials allows Hg to enter environmental waste streams [22,23]. Once released into aquatic environments, Hg can accumulate in sediments and aquatic organisms, particularly fish, increasing the likelihood of human exposure through dietary consumption and potentially causing adverse health effects with chronic exposure [24,25]. Ni and Co are potentially hazardous metals used in various industrial processes. Ni is widely used in stainless steel, alloys, electroplating, and batteries [26], while Co can co-occur with nickel in electrodeposition and coatings [27]. Excessive Co exposure has been associated with cardiovascular, endocrine, and hematological effects [28], while Ni can accumulate in sediments and aquatic organisms, creating potential human exposure pathways through contaminated fish [29]. Zn and Mn are essential micronutrients required for enzymatic function and immune competence, but excessive exposure can be toxic [30,31]. Chronic exposure to elevated Zn concentrations has been associated with gastrointestinal disturbances [32], while excessive Mn exposure through industrially contaminated drinking water can lead to brain Mn accumulation and has been associated with neurological impairment [33].

The Turag River, which flows through the northern outskirts of Dhaka, is severely affected by rapid industrialization [34]. Untreated effluents of huge volume are released directly into the river from industries such as the textile and dyeing industry at Gazipur, causing substantial environmental degradation [35]. Such pollution can increase heavy-metal accumulation in aquatic organisms, particularly fish, creating potential human exposure through prolonged consumption [36].

Despite growing evidence of heavy metal pollution in Bangladesh’s rivers, relatively few studies have examined the spatial distribution and potential sources of selected heavy metals in industrially influenced areas. Far fewer studies have gone a step further to investigate the potential sources of metals like Hg, Ni, Zn, Mn, and Co, or to assess the non-carcinogenic health risks they pose to nearby communities. This gap is particularly relevant in Gazipur, where a dense cluster of textile, dyeing, and tannery industries releases poorly treated effluents into the Turag River and surrounding water bodies. Understanding the potential sources and environmental distribution of these metals is important for informing pollution control measures and protecting the health of people living nearby. This study, therefore, aims to assess the spatial distribution of selected metals such as Hg, Ni, Zn, Mn, and Co in the Gazipur district and to explore their potential source patterns and non-carcinogenic health risks in industrial and non-industrial zones along the Turag River.

## 2. Methodology

### 2.1. Study area and sampling

The study was carried out in Gazipur District, Bangladesh, along the Turag River, with two zones having varying degrees of industrial influence. The industrial zone was identified as Kashimpur, Islampur, and Kalakoir based on the number of textile, dyeing, pharmaceutical, and metal processing industries, which are known to cause heavy metal contamination [37–39]. On the other hand, Rayerdia, NogorVela, Senpara, and Birtul were chosen as non-industrial zones because their land use is mostly agricultural and residential, and there are no major industries [40]. Both zones are linked to the Turag River system, but the non-industrial sites were not located within known industrial discharge areas or high-density urban areas, thereby reducing direct anthropogenic inputs. However, it is not possible to rule out completely the possibility of hydrological connectivity via the river system. Thus, it was deemed a relatively low-impact reference zone, not an uncontaminated background site. This distinction is important because hydrological connectivity through the Turag River may allow contaminant transport from upstream and other anthropogenic sources into the non-industrial zone.

To reduce the effects of local confounding factors, the sampling locations in both zones were chosen to ensure that environmental conditions and land use were as similar as possible, to facilitate comparison of metal levels between areas with different degrees of industrial influence. Three sampling sites were established in each zone, and within each sampling site, three sampling locations were placed about 500 m apart to provide spatially distributed observations and reduce the potential influence of closely spaced observations. Therefore, a total of 9 sampling locations were studied across each zone. One soil sample and one water sample were taken from each of the sampling locations, resulting in a total of 18 soil samples and 18 water samples. All sampling locations were recorded with a handheld Global Positioning System (GPS) and are presented in S1 Table, and the spatial distribution of sampling points is shown in Fig 1. The sampling was carried out in March 2026.

**Fig 1:**
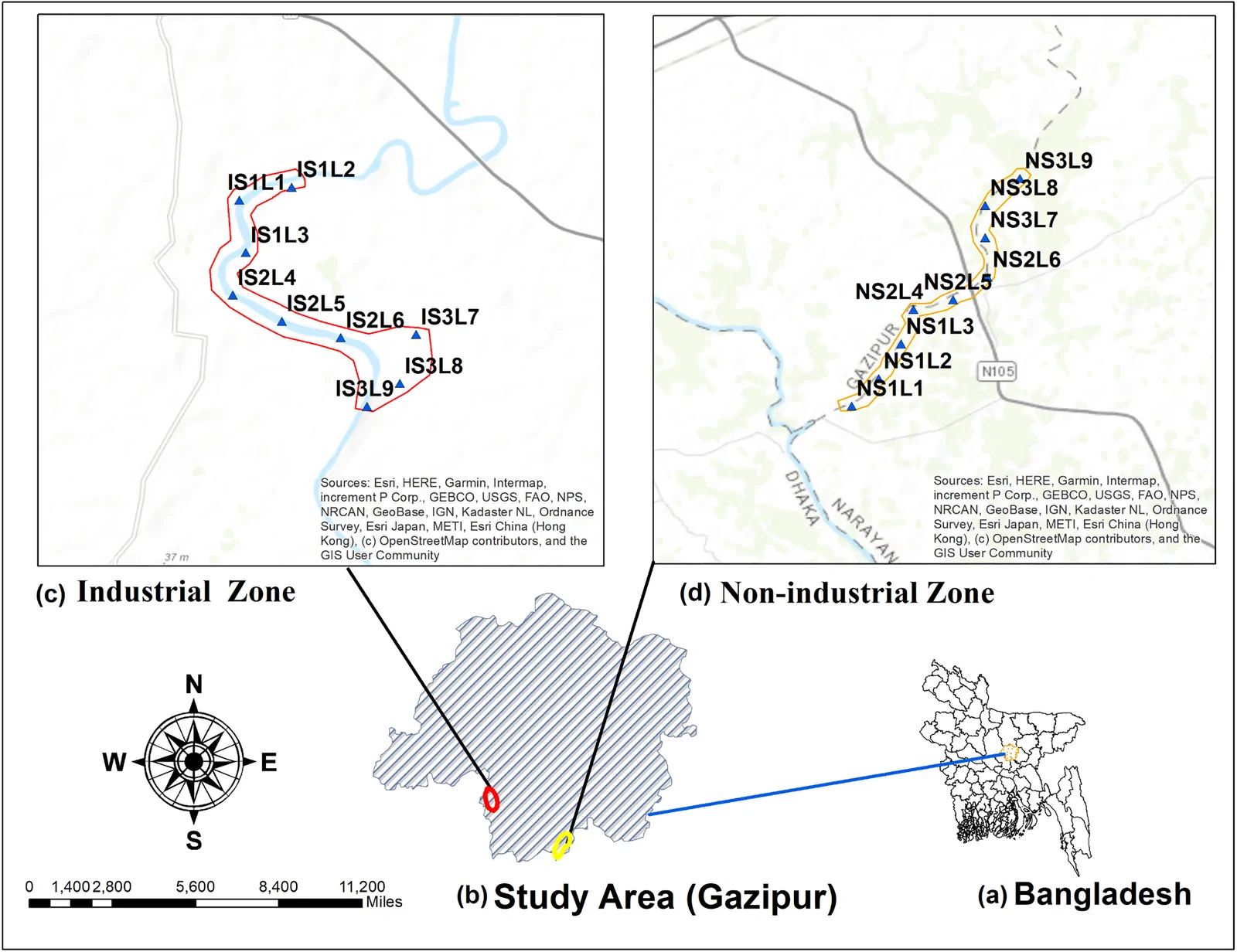
Study area map. The base map and administrative boundaries are adapted from the Humanitarian Data Exchange (https://data.humdata.org/dataset/cod-ab-bgd), used under a CC BY 4.0 License. Industrial and non-industrial boundaries are sourced from OpenStreetMap (https://www.openstreetmap.org/copyright), available under the Open Database License. All other data points were collected by the authors. This figure is original and licensed under CC BY 4.0.

### 2.2. Soil sample collection and preparation

A stainless-steel trowel was used to collect soil samples from a depth of 15–20 cm. About 500 grams of soil were taken from each sampling location. At each sampling location, 3–5 subsamples were collected from different points within a 1 m × 1 m grid, including the center and surrounding points, using approximately similar amounts of soil from each point. The subsamples were thoroughly homogenized to form one composite sample representing that sampling location. Each composite sample was analyzed separately as a representative sample of its respective sampling location. Visible grass, litter, and surface debris were removed before soil collection; no defined thickness of surface soil was removed. All the samples were stored in clean polyethylene bags, labeled, and sent to the laboratory for further processing.

Clean sampling equipment and containers were used throughout the sampling process to ensure sample quality and minimize external contamination. To remove moisture from the soil samples, the samples were air-dried in the lab with limited exposure to direct sunlight for a few days. Samples were put on clean trays and kept away from dust and other environmental contaminants during the drying period. Dried samples were gently crushed with a mortar and pestle, homogenized, and passed through a 0.5 mm stainless steel sieve. The fraction passing through the 0.5 mm sieve (<0.5 mm) was collected and used for digestion and subsequent metal analysis. Soil samples were then digested by standard methods as outlined by the US Environmental Protection Agency (USEPA) [41].

### 2.3. Water sample collection and preparation

Pre-cleaned high-density polyethylene (HDPE) bottles were used to collect about 500 mL of water from each sampling location. Each bottle was rinsed with sample water before sampling to reduce cross-contamination. 5 mL of ultrapure nitric acid (HNO_3_, 65%) was added to each bottle immediately after sampling to preserve the sample and to prevent precipitation of metals. All the samples were labeled and transported to the laboratory in insulated containers maintained at 4°C.

Contamination-free handling was maintained during sample collection, transportation, and storage to ensure sample integrity and analytical reliability. The preserved samples were stored at 4°C until analysis. The pretreatment and acid digestion of the samples were carried out by standard procedures recommended by the American Public Health Association (APHA) [42,43].

### 2.4. Metal analysis

The concentrations of Hg, Ni, Mn, Zn, and Co in the digested soil and water samples were determined using inductively coupled plasma–mass spectrometry (ICP-MS; NexION 2000, PerkinElmer, USA). The ICP Multi-Element Standard Solution XIII (Merck, Germany) was used to prepare calibration standards in the ppb concentration range. All the standards and samples were spiked with an internal standard mixture (45Sc, 73Ge, 89Y, 115In, 185Re, 193Ir, and 209Bi) before analysis to compensate for instrumental drift, matrix effects, and sample introduction variations. The levels of metals were determined by mass-to-charge ratio (m/z) and given in mg/kg for soils and mg/L for water.

### 2.5. Quality assurance and quality control

To ensure the reliability of the analytical results, quality assurance and quality control procedures were followed during sample preparation and instrumental analysis. To minimize contamination, all glassware and sample containers were carefully cleaned with ultrapure acid and deionized water before use. The instrument performance was checked with NexION Setup Solution (PerkinElmer, USA) before analysis. Calibration curves were found with a concentration range of 1–100 ppb, with the coefficients of determination (R^2^) being greater than 0.9995. The relative standard deviation (RSD) values were kept within 5–8% for each sample, and each sample was analyzed in triplicate. Certified reference materials (SRM NIST 1643f and SRM 1648a) were used to check the analytical accuracy, and satisfactory recoveries were achieved for the target metals ranging from 95.76% to 104.21%. No field blanks were collected during field sampling; however, reagent blanks and filter blanks were used to assess potential contamination during sample preparation and filtration, while spike samples were used to assess analytical accuracy. Concentrations were also adjusted for blanks and recoveries to improve data quality.

### 2.6. Non-carcinogenic health risk

The USEPA has developed a substantial exposure risk model that enables us to assess the level of potential risk to human health in different age groups within an area under investigation [44]. Exposure to pollutants through ingestion, inhalation, and dermal contact is acknowledged as a significant route that can significantly impact human health [45]. The non-carcinogenic risk was computed using equations 1–4 as suggested by USEPA [45,46].

In this study, the health-risk assessment was restricted to non-carcinogenic endpoints because the primary objective was to evaluate potential non-carcinogenic effects associated with the selected heavy metals using chronic daily intake (CDI), hazard quotient (HQ), and hazard index (HI). A carcinogenic risk assessment was not performed because it was beyond the predefined scope of the present assessment.


$$\begin{array}{c} \begin{array}{c} {CDI}_{ing}=\frac{Cn\times IngR\times EF\times ED}{BW\times AT}\times \;CF \end{array} \end{array}$$ (1)

$$\begin{array}{c} \begin{array}{c} {CDI}_{dermal}=\frac{Cn\times SA\times AF\times ABSd\times EF\times ED}{BW\times AT}\times CF \end{array} \end{array}$$ (2)

$$\begin{array}{c} \begin{array}{c} {CDI}_{inh}=\frac{Cn\times InhR\times EF\times ED}{BW\times AT\times PEF} \end{array} \end{array}$$ (3)

$$\begin{array}{c} \begin{array}{c} \sum HQ=\sum \frac{{CDI}_{i}}{{RfD}_{i}} \end{array} \end{array}$$ (4)

Where CDI_ing_, CDI_dermal_, and CDI_inh_ are the chronic daily intake from ingestion, dermal contact, and inhalation, respectively. Cn is the soil and water heavy metal concentration (mg/kg and mg/L, respectively); IngR is the ingestion rate for soil (mg/day) and water (L/day) metals; EF (day/year) and ED (year) are the exposure frequency and exposure duration, respectively; CF (kg/mg) is the conversion factor; BW (kg) and AT (year) are the average body weight and average time, respectively; SA (cm^2^) and AF (mgcm^2^) are the exposed skin area and adherence factor, respectively; ABSd is the dermal absorption factor; InhR (m^3^/day) is the inhalation rate; PEF (m^3^/kg) is particle emission factor. RfD (mg/kg/day) is the reference dose, and “i” is the number of exposure pathways. The exposure parameters and their corresponding values used in the non-carcinogenic health risk assessment are presented in S2 Table and S3 Table. An HQ or HI value above 1 indicates a potential non-carcinogenic health concern under the modeled exposure assumptions, whereas a value below 1 generally indicates a lower level of concern under those assumptions [47–49]. These indices are screening tools and do not directly establish actual exposure or adverse health effects in the study population.

### 2.7. Statistical analysis and tools used

Descriptive statistics, including mean and standard deviation (SD), were calculated using IBM SPSS Statistics version 26. Pearson correlation analysis was used to determine the relationships between the metals investigated, and principal component analysis (PCA) and hierarchical cluster analysis (HCA) were used to explore potential sources and similarities in metal distribution patterns. Given the limited number of sampling locations per zone relative to the number of metals analyzed, these multivariate analyses were considered exploratory and were used to identify preliminary patterns of association rather than provide definitive source apportionment. For PCA, eigenvalues over one were kept, and varimax rotation was used to make the analysis more interpretable. Non-carcinogenic health risk assessments, including CDI, HQ, and HI, were calculated using Microsoft Excel 2016. The study area map and the visualization of the spatial distribution of metal concentrations (both in industrial and non-industrial settings) were prepared using ArcGIS 10.7.1 (ESRI, Redlands, CA, USA). All GIS maps were independently developed by the author and generated using the projected Universal Transverse Mercator (UTM) coordinate system, Zone 45N. Statistical significance for Pearson correlation analysis was considered at p < 0.05.

## 3. Results

### 3.1. Metal concentration in soil and water

The concentrations of Hg, Ni, Mn, Zn, and Co in the soil samples differed between the industrial zone and the non-industrial zone (S4 Table). In the industrial area, mean concentrations followed a decreasing hierarchy of Mn > Zn > Ni > Co > Hg, with values of 520.84 **±** 270.37 mg/kg, 212.64 **±** 163.99 mg/kg, 181.05 **±** 237.59 mg/kg, 12.68 **±** 6.49 mg/kg, and 5.18 **±** 1.67 mg/kg, respectively. The non-industrial zone showed comparatively lower levels for most metals, with mean concentrations of 454.57 ± 137.52 mg/kg for Mn, 159.48 ± 52.97 mg/kg for Zn, 65.04 ± 22.86 mg/kg for Ni, 14.51 ± 4.32 mg/kg for Co, and 3.40 ± 1.91 mg/kg for Hg. A few industrial samples showed higher concentrations than baseline and reference values (especially Ni and Zn), suggesting very high and localized anthropogenic enrichment. In both zones, however, Hg mean levels constantly exceeded the baseline, indicating a wider regional distribution.

Heavy metal concentrations in water samples were generally low but varied spatially in both study areas (S5 Table). In the industrial zone, mean concentrations were in the order of Mn > Zn > Ni > Hg > Co, with values of 2.96 × 10^−1^ **±** 4.54 × 10^−1^ mg/L, 8.16 × 10^−2^ **±** 4.68 × 10^−2^ mg/L, 7.42 × 10^−3^ **±** 2.51 × 10^−3^ mg/L, 1.63 × 10^−3^ **±** 5.87 × 10^−4^ mg/L, and 7.11 × 10^−4^ **±** 4.31 × 10^−4^ mg/L, respectively. Conversely, the non-industrial zone displayed a modified distribution profile dominated by Zn, following the sequence of Zn (8.05 × 10^−2^ **±** 4.33 × 10^−2^ mg/L)> Mn (4.95 × 10^−2^ **±** 9.77 × 10^−3^ mg/L)> Ni (5.13 × 10^−3^ **±** 9.68 × 10^−4^ mg/L)> Hg (2.14 × 10^−3^ **±** 9.45 × 10^−4^ mg/L)> Co (4.00 × 10^−4^ **±** 1.00 × 10^−4^ mg/L). The mean concentrations of Hg and Zn were consistently high along the two zones, suggesting localized impacts from industrial effluent.

### 3.2. Spatial distribution of soil and water metals

The soil spatial distribution maps for these two zones indicated opposing accumulations associated with land uses. A common spatial concentration pattern was observed at the northern boundary in the industrial area for the contaminants: Hg, Ni, Mn, Zn, and Co. The hotspots of Hg, Mn, Zn, and Co were concentrated in the extreme north-west, around IS1L1 and IS1L2, while the Ni hotspot was slightly extended eastwards to lie exactly on the top edge at IS1L2 (Fig 2). The clear spatial association of these heavy metal concentrations in the industrial north may reflect a common anthropogenic influence associated with nearby industrial activities. The non-industrial maps, however, indicated that these metals have no common pattern, with them being separated. Hg had developed a distinct hotspot in the central part in the vicinity of NS2L5, while Ni had reached its highest value only in the west close to NS2L4. For Mn, the maximum high-level saturation was observed across the entire mapped area; therefore, a completely different trend was observed. In the meantime, Zn was spread out uniformly throughout nearly the entire area, while Co created an intense hotspot in the southwest corner, close to NS1L2 (Fig 3). The isolated and unconnected hotspots in the non-industrial zone may be consistent with diffuse and mixed influences rather than a single localized source.

**Fig 2:**
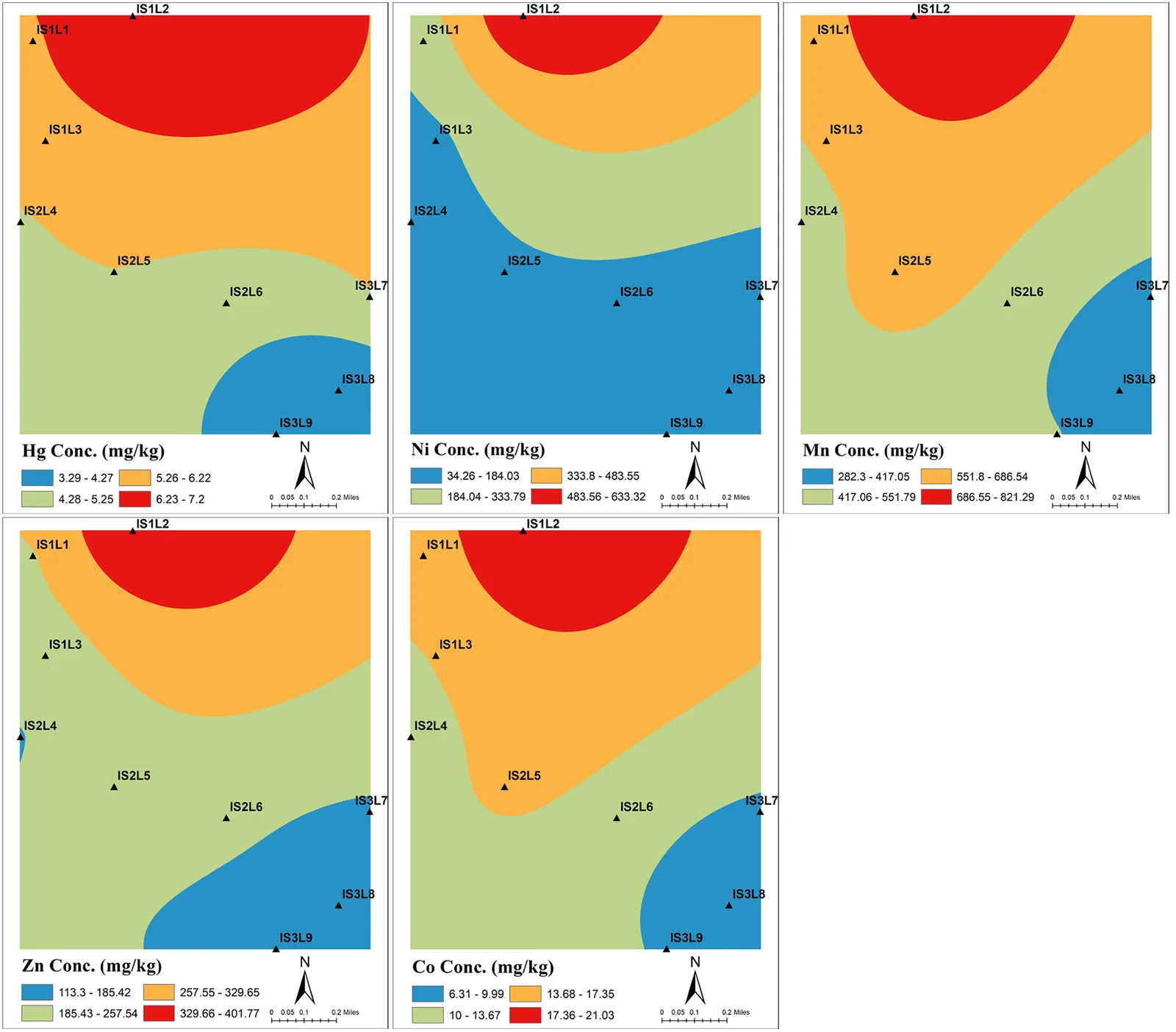
Spatial distribution of soil metals from the industrial zone.

**Fig 3:**
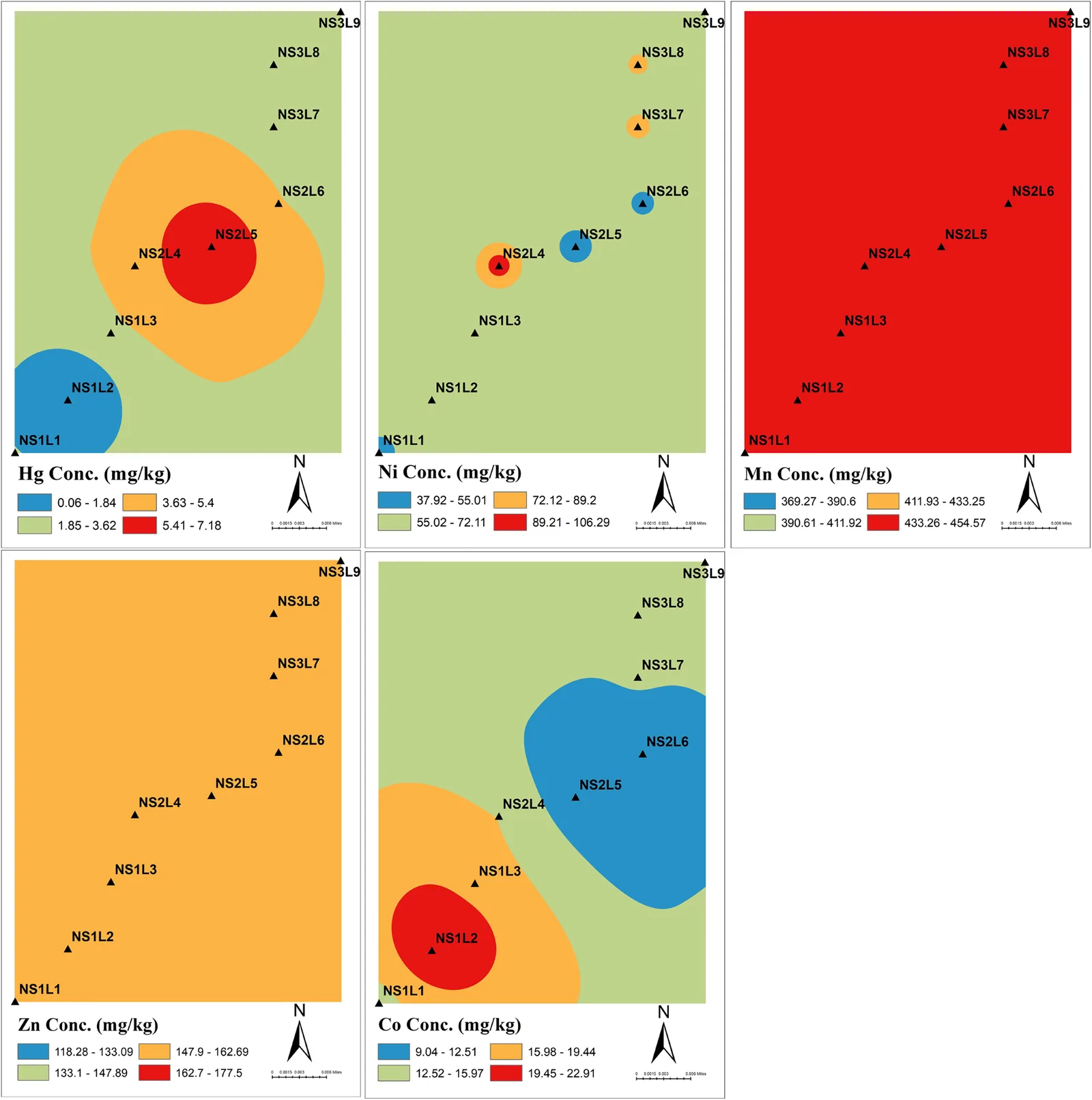
Spatial distribution of soil metals from the non-industrial zone.

For water metals, in the industrial area, the heavy metals were seen to be more concentrated at certain locations: in the north-west, at IS1L1 and IS1L2, the concentration of Hg was high; on the eastern side, the concentration of Mn was maximum at IS3L7; and Zn and Co formed isolated pollution islands in the west and north (Fig 4). These intense and distinct hotspots may reflect localized anthropogenic influence and may be associated with localized inputs from nearby industrial activities. The non-industrial area, however, showed a completely different spatial distribution pattern. Zn's distribution was bifocal, with strong hotspots in the northeast (NS3L7/NS3L8) and the west (NS2L4), whereas Hg was concentrated in the northeast, near NS3L7. At the same time, Mn exhibited a large, continuous plume in the west and central portions, while Co showed almost completely uniform saturation in the entire mapped area (Fig 5). Such a widespread and high dispersion geographic architecture may be consistent with diffuse and mixed influences, including agricultural runoff, natural lithogenic weathering, and regional atmospheric deposition, rather than a clearly identifiable localized source.

**Fig 4:**
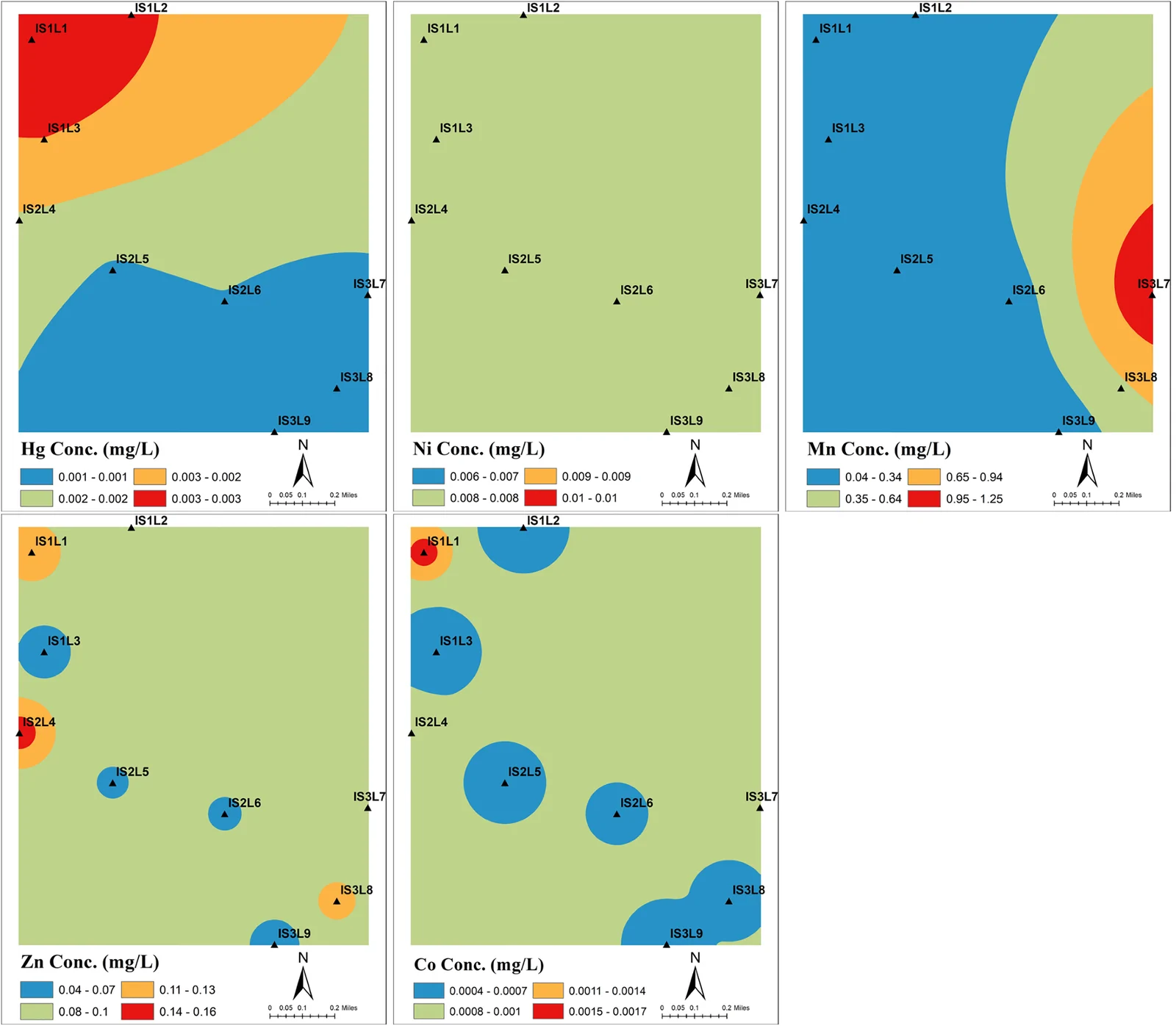
Spatial distribution of water metals from the industrial zone.

**Fig 5:**
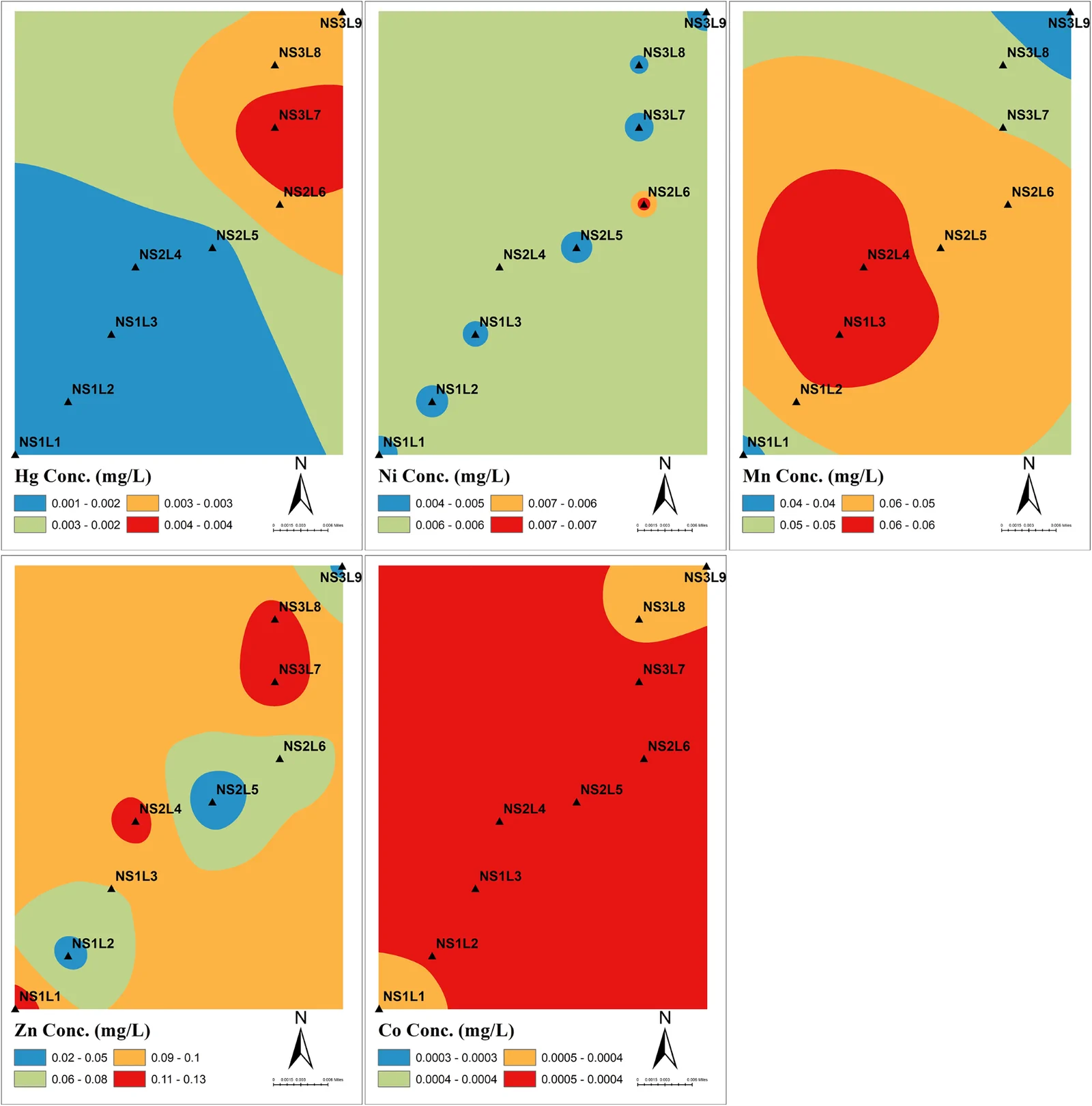
Spatial distribution of water metals from the non-industrial zone.

### 3.3. PCA of soil and water metals

In order to determine the relationships between Hg, Ni, Mn, Zn, and Co in soil and water samples from the industrial and non-industrial areas, PCA was applied. In industrial soil, only one principal component (PC1) with an eigenvalue greater than one explained 90.86% of the total variance. All metals showed strong positive loadings on PC1, including Hg (0.899), Ni (0.934), Mn (0.964), Zn (0.978), and Co (0.988). The strong positive loadings of all five metals on a single component suggest that these metals may share common sources or controlling processes in the industrial zone. Likewise, a single principal component was obtained for non-industrial soil, which explained 69.95% of the total variance. The positive loadings were strong for Mn (0.912), Zn (0.925), and Co (0.931), and moderate for Ni (0.575). However, Hg had a strong negative loading (−0.783). The distinct inverse relationship for Hg, combined with tight clusters of Mn, Zn, and Co, may indicate that soil contamination in the non-industrial zone is influenced by multiple pathways rather than a single common source.

The PCA loading plots of water metals from both zones are presented in Fig 6. For industrial water samples, 2 principal components describing 84.07% of the total variance were obtained. Following Varimax rotation, PC1 accounted for 57.15% of the variance, with high loadings on Ni (0.968), Zn (0.828), and Co (0.933), while PC2 explained 26.91% of the variance and was highly loaded with Mn (0.860) and Hg (−0.767). This separation of the components suggests that different sources or controlling processes may contribute to the observed variation in metal concentrations in the aquatic system, one of which affects the concentrations of Ni, Zn, and Co, while the other affects the concentrations of Mn and Hg. Similarly, two main components were obtained for the non-industrial water samples, accounting for 72.68% of the total variance. PC1 accounted for 47.44% of the variance and showed strong positive loadings for Ni (0.821), Mn (0.901), and Co (0.940). PC2 accounted for 25.24% of the variance and was dominated by Hg (0.874) and had a moderate loading of Zn (0.613). The multi-component nature of this pattern suggests that the observed metal variation in the non-industrial area may reflect multiple diffuse and mixed influences, such as regional agricultural runoff, natural geogenic dissolution, and localized municipal inputs.

**Fig 6:**
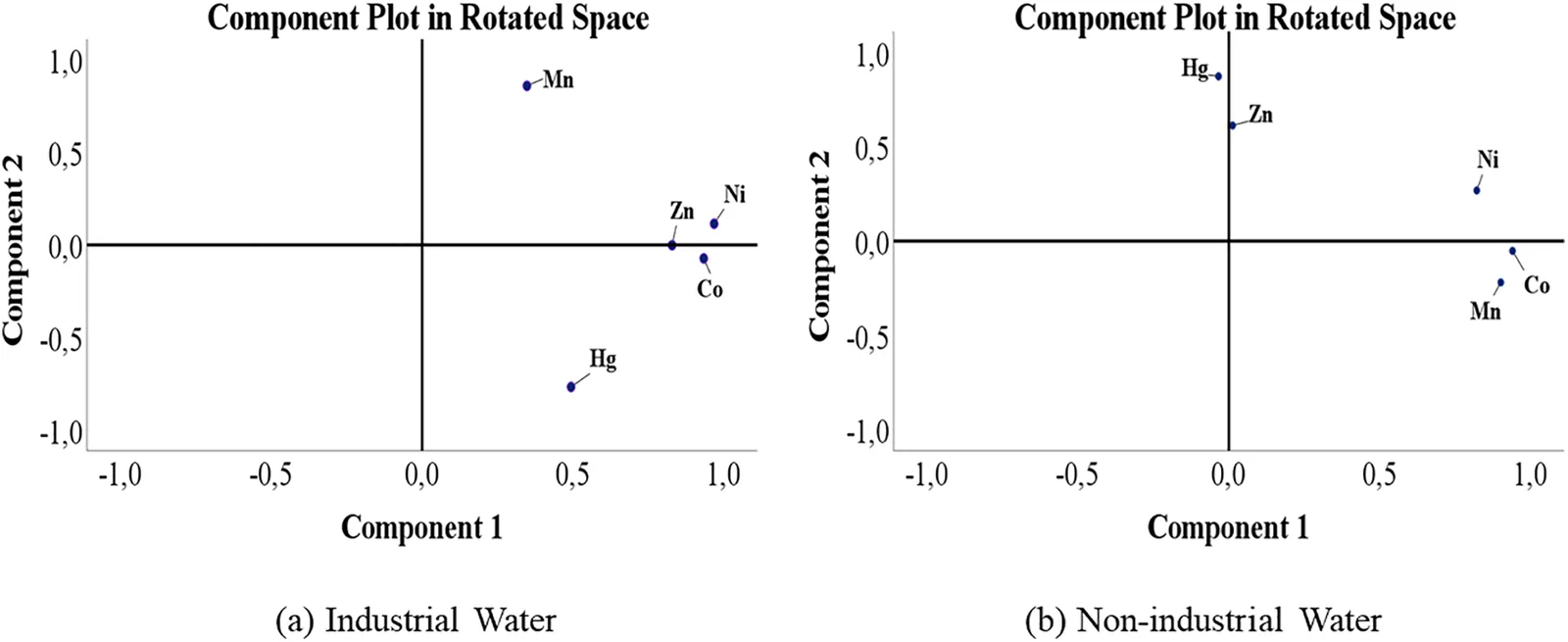
PCA loading plots of water metals from the industrial and non-industrial zones.

### 3.4. HCA of soil and water metals

The HCA was used to group the heavy metals according to their similarity in concentration patterns within each zone. The metals in the industrial soil were separated into two main clusters, with cluster 1 having a minimum linkage distance of Hg and Co, and a tight sub-group of Ni and Zn; Mn was seen as an isolated metal in cluster 2 (Fig 7a). This clustering indicates a strong association among Hg, Co, Ni, and Zn and may reflect shared sources or controlling processes. The non-industrial soil, on the other hand, exhibited a different grouping pattern, with Hg and Ni forming an independent pattern before being grouped with Co and Zn at a greater distance, while Mn remained separated (Fig 7c). This structural change may indicate that metal accumulation in the non-industrial area is influenced by multiple rather than a single common source.

**Fig 7:**
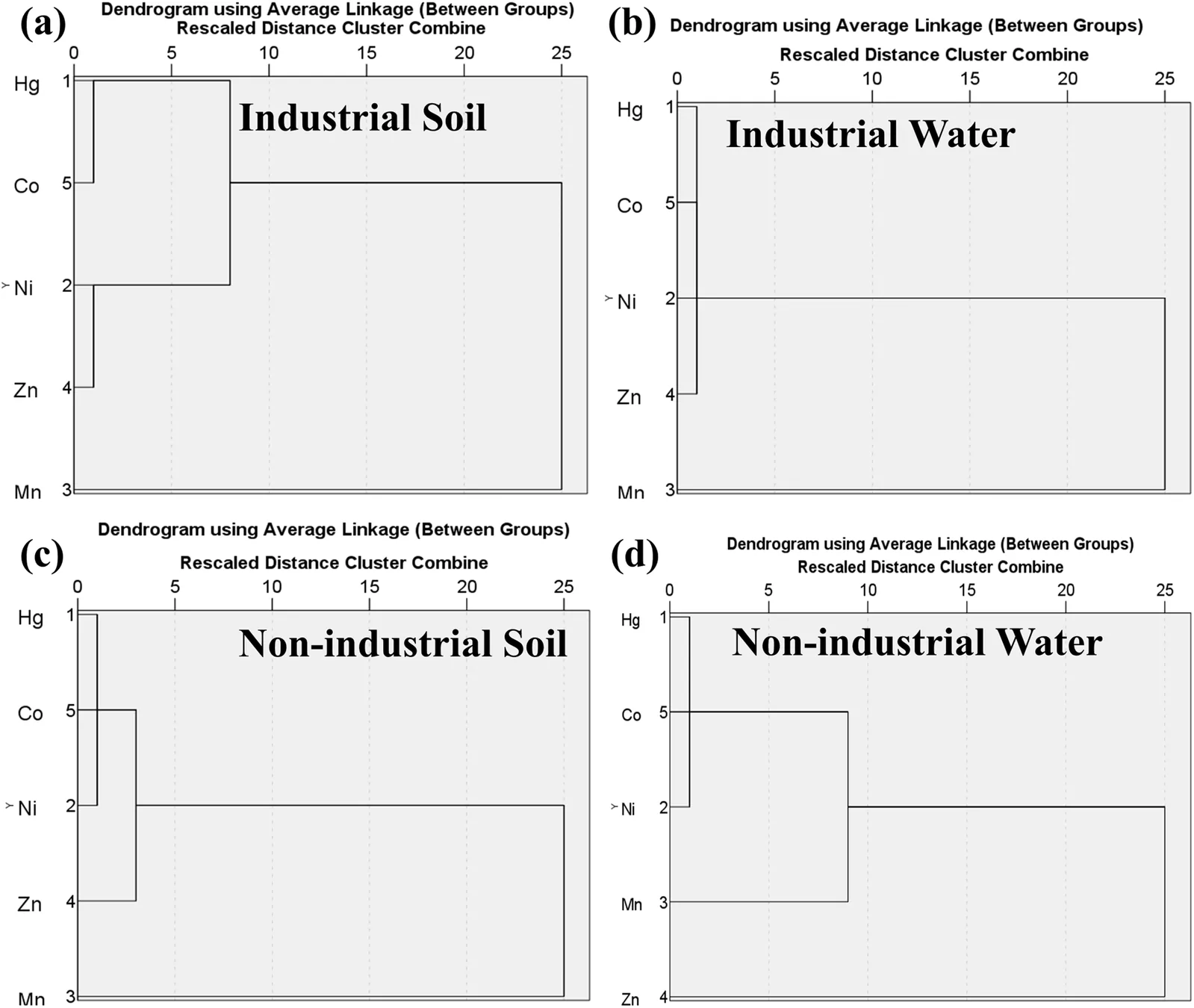
Dendrogram of soil and water metals from the industrial and non-industrial zones using hierarchical cluster analysis (HCA).

The HCA identifies different patterns of aquatic pollution between the two study areas for water samples. In the industrial water, a strong association between Hg, Co, Ni, and Zn was observed at baseline distance, whereas Mn formed a completely separate cluster that only overlapped with the first at the maximum rescaled distance (Fig 7b). This heavy separation of clusters may reflect differences in the controlling sources or environmental behavior of the metals. On the other hand, non-industrial water had a gradual ladder-like clustering sequence in which Hg, Co, and Ni cluster first, followed by Mn, and were completely separate from Zn (Fig 7d). This progressive clustering behavior may reflect the mixing and diffusion of multiple inputs, potentially including agricultural run-off and natural weathering of lithogenic materials.

### 3.5. Correlation of heavy metal levels in soil and water metals

The correlation matrices clearly showed differences in metal-to-metal relationships by land use and environmental media. For all five heavy metals (Hg, Ni, Mn, Zn, and Co), industrial soil showed a strong positive correlation, with r values ranging from 0.756 to 0.99, which were statistically significant (Fig 8a). This close elemental association implies a dominant anthropogenic point source, in which the metals are co-released on the local terrain by industrial activities. On the other hand, the non-industrial soil exhibited a reverse pattern of correlation: Hg correlates strongly negatively with Zn (−0.720) and Co (−0.702); Mn exhibited a strong positive association with Zn (0.731) and Co (0.812) (Fig 8c). This clear divide suggests that the soil metal levels in the non-industrial part are possibly influenced by different, independent pathways, which would imply that Mn, Zn, and Co come either from agricultural inputs or from natural soil weathering processes, while Hg might have another source of origin.

**Fig 8:**
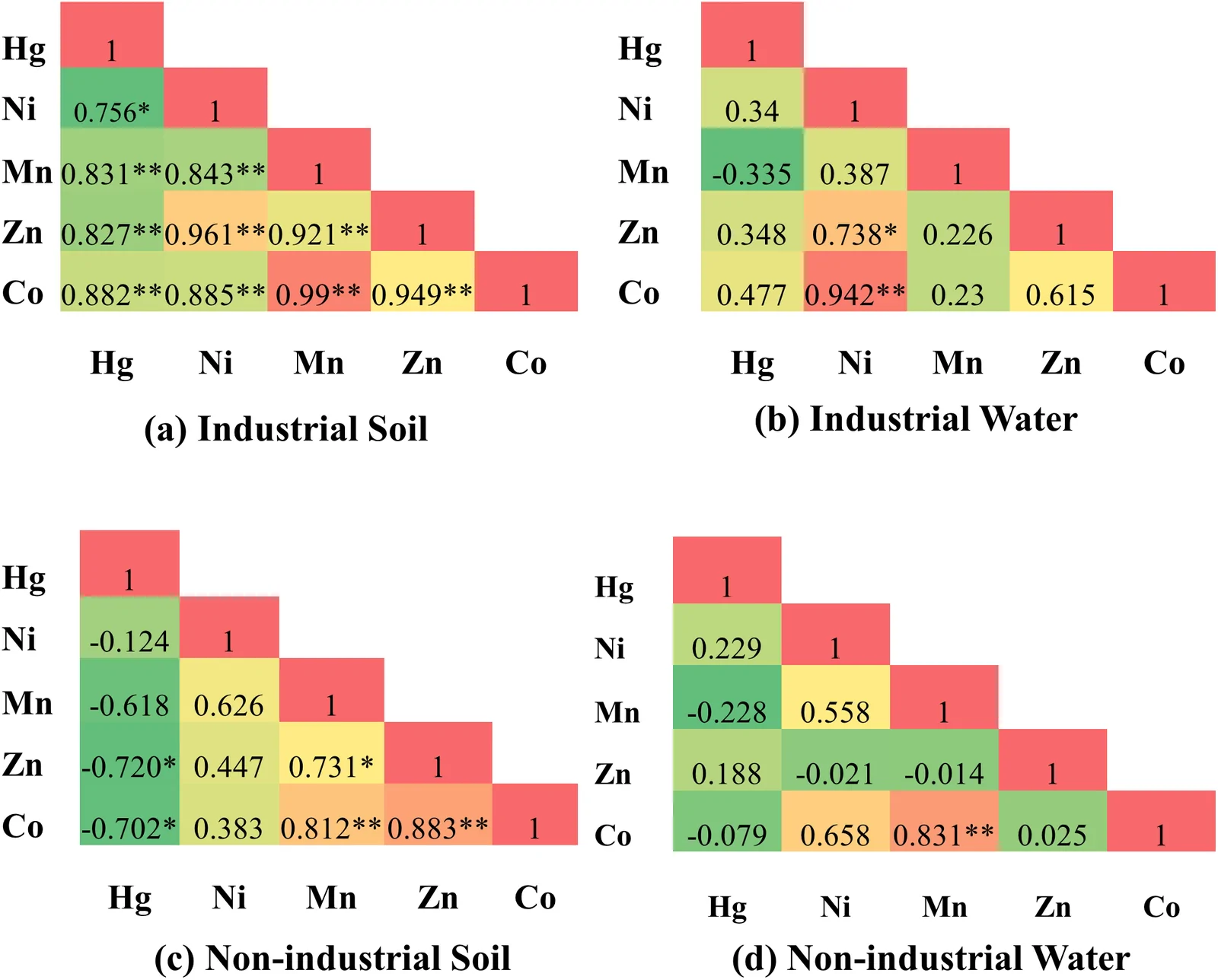
Heavy metal inter-elemental correlation coefficients in water and soil samples from the industrial and non-industrial zones. Values represent Pearson correlation coefficients (r). *p < 0.05 and **p < 0.01 (2-tailed).

The correlation values of the water samples are specific to the different aquatic transport properties in the two areas. Concerning the industrial water, the strong multi-metal bonds in the soil were no longer present. Only a strong positive correlation between Ni and Co (0.942) and a strong positive correlation between Ni and Zn (0.738) were obtained (Fig 8b). The specific linear correlation is correlated to a specific industrial waste stream in which Ni, Co, and Zn may be released together. In contrast, there are weak or negative correlations for Hg and Mn, indicating that these metals are released into the aquatic system separately. The non-industrial water, on the other hand, showed almost no correlation between most elements, except for a single and highly significant positive correlation between Mn and Co (0.831) (Fig 8d). This single strong association may be due to natural geogenic weathering, since Manganese and Cobalt are often associated in the area's bedrock and groundwater. The others do not show any correlation and may be added by diffuse, unrelated activities.

### 3.6. Non-carcinogenic risks from soil and water metals

The CDI values for soils showed that ingestion was the major exposure route for both adults and children, with dermal absorption and inhalation having relatively minor contributions. In general, the modeled soil CDI estimates were higher for children than adults in both industrial and non-industrial areas. The investigated metals showed the highest CDI values for Mn, followed by Zn and Ni (Supplementary S6 Table). The HQ analysis showed that HQ values for all metals and exposure pathways were below 1, except for Ni in children in the industrial zone. In the industrial zone, the modeled HQ for Ni in children exceeded the screening benchmark of 1 (HQ = 1.66), indicating a potential non-carcinogenic risk. Likewise, in the industrial and non-industrial zones, the children’s cumulative hazard index (HI) was over 1 (2.32 and 1.31, respectively), while the adult HI values were below 1 (Table 1). These results indicate potential non-carcinogenic concerns under the modeled soil-exposure scenarios, but do not establish actual child exposure or adverse health outcomes.

**Table 1:** Hazard quotient (HQ) and hazard index (HI) of heavy metal-contaminated soil from both industrial and non-industrial zones.

Zones	Pathways	Adult					Child				
		Hg	Ni	Mn	Zn	Co	Hg	Ni	Mn	Zn	Co
**Industrial**	***HQ***_***ing***_	2.36 × 10^−3^	1.77 × 10^−1^	5.10 × 10^−3^	9.71 × 10^−4^	5.79 × 10^−2^	2.21 × 10^−2^	1.65	4.76 × 10^−2^	9.06 × 10^−3^	5.40 × 10^−1^
	***HQ***_***dermal***_	1.35 × 10^−3^	5.50 × 10^−4^	5.08 × 10^−4^	1.55 × 10^−5^	1.39 × 10^−5^	5.04 × 10^−3^	2.06 × 10^−3^	1.90 × 10^−3^	5.80 × 10^−5^	5.19 × 10^−5^
	***HQ***_***inh***_	–	–	7.34 × 10^−3^	5.71 × 10^−7^	4.47 × 10^−4^	–	–	3.42 × 10^−2^	2.67 × 10^−6^	2.09 × 10^−3^
	***HQ***_***total***_	3.71 × 10^−3^	1.78 × 10^−1^	1.29 × 10^−2^	9.87 × 10^−4^	5.84 × 10^−2^	2.71 × 10^−2^	1.66*	8.37 × 10^−2^	9.12 × 10^−3^	5.42 × 10^−1^
	***HI***	**Adult**					**Child**				
		2.54 × 10^−1^					2.32*				
**Non-industrial**	***HQ***_***ing***_	1.55 × 10^−3^	6.36 × 10^−2^	4.45 × 10^−3^	7.28 × 10^−4^	6.62 × 10^−2^	1.45 × 10^−2^	5.94 × 10^−1^	4.15 × 10^−2^	6.80 × 10^−3^	6.18 × 10^−1^
	***HQ***_***dermal***_	8.84 × 10^−4^	1.97 × 10^−4^	4.44 × 10^−4^	1.16 × 10^−5^	1.59 × 10^−5^	3.31 × 10^−3^	7.39 × 10^−4^	1.66 × 10^−3^	4.35 × 10^−5^	5.94 × 10^−5^
	***HQ***_***inh***_	–	–	6.40 × 10^−3^	4.28 × 10^−7^	5.12 × 10^−4^	–	–	2.99 × 10^−2^	2.00 × 10^−6^	2.39 × 10^−3^
	***HQ***_***total***_	2.43 × 10^−3^	6.38 × 10^−2^	1.13 × 10^−2^	7.40 × 10^−4^	6.68 × 10^−2^	1.78 × 10^−2^	5.95 × 10^−1^	7.31 × 10^−2^	6.84 × 10^−3^	6.21 × 10^−1^
	***HI***	**Adult**					**Child**				
		1.45 × 10^−1^					1.31*				

*Potential non-carcinogenic risk

The dermal route made a greater contribution to the total CDI than the ingestion route in both the industrial and non-industrial zones for water exposure. Like soil exposure, children had higher CDI scores than adults. It was found that, of the metals studied, the highest exposure was for Mn in both zones (Supplementary S7 Table). However, for each metal, as well as for the cumulative HI values for adults and children, all values were well below the acceptable level of 1 (Supplementary S8 Table). The modeled water-based estimates did not indicate potential non-carcinogenic concern under the assumed exposure scenarios, as all HQ and HI values were below 1, although the modeled CDI values for children were relatively higher than those for adults.

## 4. Discussion

The present study provides a comprehensive assessment of heavy metal contamination in soil and surface water from the Turag River area of the Gazipur district, Bangladesh. Overall, the findings demonstrate that industrial activities substantially influence the distribution and accumulation of heavy metals, particularly in soil, and non-industrial areas are not free from contamination. Although industrial sites generally exhibited higher concentrations of Hg, Ni, Mn, and Zn than non-industrial locations, several metals were also detected at appreciable levels in the latter. Importantly, the modeled soil-risk estimates indicated the greatest potential non-carcinogenic concern for children in the industrial zone, with Ni showing an HQ above 1.

The consistent clustering of Hg, Ni, Mn, Zn, and Co on a single principal component explaining over 90% of variance in industrial soil, together with their tight co-clustering in HCA and strong pairwise correlations (r = 0.756–0.99), suggests that these metals may share common sources or controlling processes, potentially including effluent or particulate inputs associated with nearby industrial activities such as electroplating, chemical manufacturing, or textile dyeing. In contrast, the inverse loading of Hg against Mn, Zn, and Co in non-industrial soil, and the near-absence of inter-metal correlation in non-industrial water show that contamination away from the industrial belt arises from several independent, low-intensity pathways, including agricultural inputs, municipal waste, traffic emissions, and geogenic weathering. Practically, these screening results support further investigation of potential sources contributing to the elevated Ni HQ in children in the industrial zone, while the HI values above 1 in both zones warrant broader assessment of the combined non-carcinogenic concerns under the modeled exposure assumptions. The finding suggests that local exposure to these surface waters is occurring mainly through bathing, washing, or agricultural contact activities rather than direct drinking, and risk communication and mitigation measures should be tailored accordingly.

This research highlighted that most metals exhibited greater concentrations in the industrial area relative to the non-industrial area. This could be attributed to various secondary sources, including traffic emissions, municipal waste disposal, and localized activities. Furthermore, atmospheric deposition and the movement of contaminated sediments via the Turag River could lead to the redistribution of heavy metals beyond the immediate vicinity of industrial areas. Thus, the non-industrial zone should be interpreted as a relative reference area rather than a pristine background site because hydrological connectivity may contribute to contaminant transfer. Various studies carried out globally across various industrial regions have revealed elevated pollution levels from a range of metals, along with associated health risks, which highlighted the greater risk for people who lived near industry [40,50–53].

Among the metals investigated, Mn was the most abundant element in soil samples from both industrial and non-industrial zones, followed by Zn, Ni, Co, and Hg. Mn is the 12th most abundant element on Earth and ranks as the fifth most abundant metal [54]. Annually, natural earth erosion contributes significant amounts of manganese into the atmosphere, soil, and waterways, making it available for uptake by microbes, plants, and animals [55]. Manganese compounds have major applications in the production of steel, manufacture of batteries, welding operations, pigments, and alloy sectors [56,57], which are very common industrial activities within the Gazipur region. The elevated Mn concentrations therefore likely reflect the combined influence of lithogenic sources and industrial discharge. Industrial areas worldwide show elevated Mn, Hg, Ni, Co, and Zn in soils, with concentrations varying by industry type, proximity to emission sources, and local geology. For example, studies conducted in China’s urban industrial zones showed Mn reached the highest mean concentration among studied metals [58,59]. Moreover, a global meta-analysis of 174 cities reported mean soil concentrations of Hg, Co, Ni, and Zn, all exceeding their average crustal values [60].

Our model-based estimates indicated higher potential non-carcinogenic risk for children than adults, consistent with findings from other metal risk-assessment studies. A study conducted in agricultural soils around Sinop Province, Turkey, similarly reported non-carcinogenic risk above the safety threshold for children while remaining low for adults [61]. Likewise, a study in Sialkot, Pakistan, found that children are 4–5 times more vulnerable to health risks than adults according to hazard index values [62]and children bore disproportionately higher non-carcinogenic risk than adults [63]. This pattern, replicated across very different exposure media and geographic settings, reflects children’s higher soil ingestion rate and lower body weight relative to adults in USEPA exposure models, and reinforces that our result is not an artifact specific to Gazipur.

Our findings suggest a common anthropogenic source influence on the industrial zone soil and water contaminants, consistent with previous studies that traced elevated soil and water heavy metal burdens in the tannery-industrial belt of Savar, Dhaka, to concentrated industrial discharge [63,64]and another study reported that irrigation water and soil in the multi-industry zones of Gazipur District (Banglabazar, Kashimpur, and Chandra) were contaminated well above permissible limits [53,65], attributable to nearby small-scale factories. A previous study on the Turag River area in Gazipur District found that heavy metals like arsenic (As), lead (Pb), cadmium (Cd), chromium (Cr), and copper (Cu) were in greater concentration in the industrial areas compared to the non-industrial areas, although they did not count the metals like Hg, Ni, Mn, Zn, and Co that are included in our study [40].

However, our results diverge from several of these Bangladeshi tannery-belt studies in the identity of the metal driving risk: whereas Cr is the overwhelming contaminant and principal non-carcinogenic and carcinogenic risk driver in Hazaribagh and Savar soils [63,64,66]. This difference likely reflects the specific mix of industries operating in our industrial zone, which may be dominated by metal-finishing or electroplating operations that discharge Ni rather than the chromium-intensive tanning processes that dominate the Hazaribagh and Savar tannery clusters, underscoring that source-specific, rather than generic, metal panels are needed when assessing industrial risk in Bangladesh. Our finding that ingestion, rather than inhalation, was consistently the major soil exposure pathway is consistent with a previous study that reported that 97.8–99.9% of soil-related exposure occurred via oral ingestion [66]and a national-scale Chinese survey found ingestion-driven hazard quotients dominating total risk [67]. Besides, consistent with our findings, studies conducted in India [68] and in China [69,70] found ingestion to be the main pathway of causing health risk to the population. However, previous studies carried out in Bangladesh [71] and in other countries worldwide, such as in Iran [72] and in Egypt [73], reported dermal exposure to water metals to be the main pathway of causing non-carcinogenic risk.

This study has several limitations. First, sampling was conducted at a single time point, a cross-sectional snapshot, and cannot capture seasonal variability or account for the potential short-term influence of antecedent precipitation on metal concentrations. Studies have shown that irrigation water, soil, and vegetable metal concentrations in nearby Gazipur industrial areas differ substantially between dry and wet seasons due to rainfall dilution, and similar seasonal fluctuation cannot be ruled out at our sites [65]. Second, the metal panel was restricted to five elements (Hg, Ni, Mn, Zn, and Co), and other potentially toxic metals of concern, including Cr, Pb, Cd, and As, were not analyzed; therefore, the findings represent risks associated with the selected metals rather than the full heavy-metal burden. Third, the relatively small sample size, with nine sampling locations per zone, limits the statistical power and generalizability of the findings and constrains the precision of the interpolated contamination maps and the robustness of the multivariate analyses. Moreover, exact distances from individual sampling locations to nearby industrial facilities and other local features were not recorded during the field survey. The relatively small number of observations per zone also limits the robustness of PCA and HCA; therefore, these analyses should be regarded as exploratory rather than definitive source-apportionment approaches. Similarly, the interpolated maps should be interpreted as indicative spatial representations rather than high-precision predictions. Soil was collected at 15–20 cm depth after removing visible grass, litter, and surface debris, without removing a defined thickness of surface soil. No specific rationale linking this sampling depth to children’s soil-contact exposure was documented; therefore, the measured concentrations may not represent ground-surface soil encountered by children. The analyzed concentrations represent the bulk-soil fraction passing through a 0.5 mm sieve (<0.5 mm), which was not separated into finer particle-size classes or characterized for particle-size-specific metal content, airborne-dust relevance, or oral bioaccessibility. Fourth, this study assessed only soil and water; sediment, locally grown vegetables, and fish were not sampled, so bioaccumulation and dietary exposure pathways were not captured, and the estimated risks may not reflect the overall heavy-metal exposure burden. Finally, the CDI, HQ, and HI calculations relied on standard USEPA default exposure parameters (e.g., body weight, ingestion rate, exposure duration) rather than population-specific behavioral or dietary data for Gazipur residents, which may over- or underestimate the actual exposure of the local population. Although Bangladesh-specific evidence is available for some individual exposure parameters, comparable validated local data were not available across all relevant exposure pathways and age groups, including exposure frequency, exposure duration, and dermal contact parameters. Therefore, the use of generic exposure assumptions introduces uncertainty into the estimated CDI, HQ, and HI values and may affect the magnitude and interpretation of the estimated risks. The estimated CDI, HQ, and HI values should therefore be interpreted as screening-level, model-based estimates under the assumed exposure scenarios and environmental conditions observed during the study period, rather than as direct measurements of population-level risk or representative estimates of year-round or long-term exposure.

Despite these limitations, the findings have some public health implications. First, the soil-based screening estimates indicated HI values above 1 for children in both zones, warranting further investigation and environmental monitoring, particularly in the industrial zone. Second, children living in or attending schools within the industrial zone may be considered a priority population for environmental health surveillance, particularly in areas with continued industrial exposure. Third, given that dermal contact was the principal water exposure route, community advisories on limiting prolonged bathing or washing contact with visibly industry-adjacent surface water bodies would be a low-cost interim protective measure while longer-term remediation is pursued.

Future research should incorporate repeated, seasonal sampling to capture temporal variability in contamination and risk; expand the metal panel to include other potentially toxic metals of public health concern; incorporate sediment, locally consumed vegetables, and fish to evaluate bioaccumulation and dietary exposure pathways alongside soil and water; collect population-specific information on exposure behaviors, such as soil ingestion, water-contact frequency, and exposure duration; conduct future sampling of ground-surface soil and analyze finer particle-size fractions and, where feasible, oral bioaccessibility; and, where sufficient local data are available, apply probabilistic or sensitivity analyses to quantify the influence of exposure-parameter uncertainty on CDI, HQ, and HI estimates, and conduct direct biological monitoring (e.g., blood or urine metal levels) in children residing near the industrial zone to validate the modeled exposure estimates against measured body burden.

## 5. Conclusion

Based on the conditions observed in this study, heavy metal contamination and modeled non-carcinogenic risk estimates were generally higher in the industrial zone than in the non-industrial zone. Soil-based screening estimates indicated potential non-carcinogenic concern for children, particularly regarding Ni in the industrial zone. These estimates are preliminary and are based on the soil fraction passing through a 0.5 mm sieve (<0.5 mm) collected at 15–20 cm depth; because finer particle-size fractions and their metal contents were not separately characterized, the estimates should not be interpreted as direct measurements of children’s actual exposure through hand-to-mouth contact or inhalation. Environmental and public health authorities should prioritize source control at the identified industrial hotspot and consider child-focused monitoring of heavy metal exposure, while recognizing that repeated seasonal assessments are needed to determine the persistence and longer-term magnitude of these risks.

## Supporting information

S1 Table Coordinates of the soil and water sampling locations. (DOCX)

S2 Table Parameters for human exposure and health risk assessment [45,47,74,75]. (DOCX)

S3 Table Reference dose (RfD) (mg/kg/day) values used in this study [76–80]. (DOCX)

S4 Table Metal concentration (mg/kg) in soil samples collected from both the industrial and non-industrial zones (baseline and reference values [81]). (DOCX)

S5 Table Metal concentration (mg/L) in water samples collected from both the industrial and non-industrial zones (baseline and reference values [81]). (DOCX)

S6 Table Chronic daily intake (CDI) of heavy metal-contaminated soil from the industrial and non-industrial zones through ingestion, dermal absorption, and inhalation (mg/day). (DOCX)

S7 Table Chronic daily intake (CDI) of heavy metal-contaminated water from the industrial and non-industrial zones through ingestion, dermal absorption, and inhalation (mg/day). (DOCX)

S8 Table Hazard quotient (HQ) and hazard index (HI) of heavy metal-contaminated water from both industrial and non-industrial zones. (DOCX)

## Abbrevations

ICP-MS: Inductively coupled plasma-mass spectrometry
GPS: Global Positioning System
USEPA: US Environmental Protection Agency
APHA: American Public Health Association
PCA: Principal Component Analysis
HCA: Hierarchical Cluster Analysis
CDI: Chronic daily intake
HQ: Hazard quotient
HI: Hazard index
HNO_3_: Nitric acid
Hg: Mercury
Ni: Nickel
Zn: Zinc
Mn: Manganese
Co: Cobalt
