# Supplementary material for: Source apportionment and non-carcinogenic health risk assessment of selected heavy metals in the Turag river area of Gazipur District, Bangladesh
Source: PLoS One. 2026 Nov 6;21(11):e0362351. doi: 10.1371/journal.pone.0362351 (PMC13742873; doi:10.1371/journal.pone.0362351)
Supplement: Supplementary file 1 [file pone.0362351.s001.docx]

**S1 Table.** Coordinates of the soil and water sampling locations.

| **Zones** | **Sampling Sites** | **Sampling Locations** | **Latitude** | **Longitude** | **Soil** | **Water** |
| --- | --- | --- | --- | --- | --- | --- |
| **Industrial** | **Site 1** | **IS1L1** | 23°59′35″N | 90°19′38″E |  |  |
|  |  | **IS1L2** | 23°59′39″N | 90°19′54″E |  |  |
|  |  | **IS1L3** | 23°59′19″N | 90°19′40″E |  |  |
|  | **Site 2** | **IS2L4** | 23°59′06″N | 90°19′36″E |  |  |
|  |  | **IS2L5** | 23°58′58″N | 90°19′51″E |  |  |
|  |  | **IS2L6** | 23°58′53″N | 90°20′09″E |  |  |
|  | **Site 3** | **IS3L7** | 23°58′54″N | 90°20′32″E |  |  |
|  |  | **IS3L8** | 23°58′39″N | 90°20′27″E |  |  |
|  |  | **IS3L9** | 23°58′32″N | 90°20′17″E |  |  |
| **Non-industrial** | **Site 1** | **NS1L1** | 23°53′11″N | 90°28′04″E |  |  |
|  |  | **NS1L2** | 23°53′22″N | 90°28′15″E |  |  |
|  |  | **NS1L3** | 23°53′36″N | 90°28′24″E |  |  |
|  | **Site 2** | **NS2L4** | 23°53′50″N | 90°28′29″E |  |  |
|  |  | **NS2L5** | 23°53′54″N | 90°28′45″E |  |  |
|  |  | **NS2L6** | 23°54′03″N | 90°28′59″E |  |  |
|  | **Site 3** | **NS3L7** | 23°54′19″N | 90°28′58″E |  |  |
|  |  | **NS3L8** | 23°54′32″N | 90°28′58″E |  |  |
|  |  | **NS3L9** | 23°54′43″N | 90°29′12″E |  |  |
