# Supplementary material for: Source apportionment and non-carcinogenic health risk assessment of selected heavy metals in the Turag river area of Gazipur District, Bangladesh
Source: PLoS One. 2026 Nov 6;21(11):e0362351. doi: 10.1371/journal.pone.0362351 (PMC13742873; doi:10.1371/journal.pone.0362351)
Supplement: Supplementary file 2 [file pone.0362351.s002.docx]

**S2 Table.** Parameters for human exposure and health risk assessment [45,47,74,75].

| **Parameters** | **Units** | **Value (Resident)** | |
| --- | --- | --- | --- |
|  |  | **Adult** | **Child** |
| Ingestion rate (IngR) | Soil = mg/day  Water = L/day | Soil = 100 | Soil = 200 |
|  |  | Water = 2.2 | Water = 1.8 |
| Exposure frequency (EF) | day/year | 350 | 350 |
| Exposure duration (ED) | year | 30 | 6 |
| Average body weight (BW) | kg | 70 | 15 |
| Average time (AT = 365xED) | year | 10950 | 2190 |
| Conversion factor (CF) | kg/mg | 1.00×10^-6^ | 1.00×10^-6^ |
| Exposed skin area (SA) | cm^2^ | 5700 | 1600 |
| Adherence factor (AF) | mgcm^-2^ | 0.07 | 0.2 |
| Dermal absorption factor (ABS_d_) | unitless | 0.001 for all metals and for As =0.03 | |
| Inhalation rate (InhR) | m^3^/day | 20 | |
| Particle emission factor (PEF) soil to air | m^3^/kg | 1.36×10^-9^ | |
