# Supplementary material for: Source apportionment and non-carcinogenic health risk assessment of selected heavy metals in the Turag river area of Gazipur District, Bangladesh
Source: PLoS One. 2026 Nov 6;21(11):e0362351. doi: 10.1371/journal.pone.0362351 (PMC13742873; doi:10.1371/journal.pone.0362351)
Supplement: Supplementary file 3 [file pone.0362351.s003.docx]

**S3 Table.** Reference dose (RfD) (mg/kg/day) values used in this study [76–80].

| **Metal** | **RfD_ing_** | **RfD_inh_** | **RfD_dermal_** |
| --- | --- | --- | --- |
| Hg | 3.00×10^-4^ | - | 2.10×10^-4^ |
| Ni | 1.40×10^-3^ | - | 1.80×10^-3^ |
| Mn | 1.40×10^-1^ | 1.43×10^-5^ | 5.60×10^-3^ |
| Zn | 3.00×10^-1^ | 7.50×10^-2^ | 7.50×10^-2^ |
| Co | 3.00×10^-4^ | 5.71×10^-6^ | 5.00×10^-3^ |
