# Supplementary material for: Source apportionment and non-carcinogenic health risk assessment of selected heavy metals in the Turag river area of Gazipur District, Bangladesh
Source: PLoS One. 2026 Nov 6;21(11):e0362351. doi: 10.1371/journal.pone.0362351 (PMC13742873; doi:10.1371/journal.pone.0362351)
Supplement: Supplementary file 4 [file pone.0362351.s004.docx]

**S4 Table.** Metal concentrations (mg/kg) in soil samples collected from both the industrial and non-industrial zones (baseline and reference values [81]).

| **Zones** | **Sampling Sites** | **Sampling Locations** | **Hg** | **Ni** | **Mn** | **Zn** | **Co** |
| --- | --- | --- | --- | --- | --- | --- | --- |
| **Industrial** | **Site 1** | **IS1L1** | 5.92 | 168.13 | 514.39 | 183.69 | 13.19 |
|  |  | **IS1L2** | 8.04 | 798.33 | 1062.11 | 607.1 | 26.31 |
|  |  | **IS1L3** | 5.7 | 96.98 | 555.75 | 145.45 | 13.01 |
|  | **Site 2** | **IS2L4** | 4.92 | 110.88 | 393.28 | 149.96 | 10.6 |
|  |  | **IS2L5** | 5.4 | 165.32 | 694.98 | 239.06 | 16.06 |
|  |  | **IS2L6** | 4.58 | 145.4 | 552.98 | 246.95 | 13.01 |
|  | **Site 3** | **IS3L7** | 6.03 | 94.54 | 343.34 | 186.93 | 9.51 |
|  |  | **IS3L8** | 1.86 | 6.49 | 61.16 | 2.82 | 1.67 |
|  |  | **IS3L9** | 4.13 | 43.42 | 509.59 | 151.78 | 10.75 |
|  | **Mean ± SD** | | 5.18 ± 1.67 | 181.05 ± 237.59 | 520.84 ± 270.37 | 212.64 ± 163.99 | 12.68 ± 6.49 |
| **Non-industrial** | **Site 1** | **NS1L1** | 1.94 | 37.92 | 369.27 | 138.55 | 13.91 |
|  |  | **NS1L2** | 0.04 | 67.58 | 670.42 | 263.99 | 23.32 |
|  |  | **NS1L3** | 3.17 | 68.82 | 460.26 | 189.28 | 18.05 |
|  | **Site 2** | **NS2L4** | 4.43 | 108.87 | 564.19 | 153.87 | 15.91 |
|  |  | **NS2L5** | 7.19 | 36.97 | 267.03 | 102.96 | 10.13 |
|  |  | **NS2L6** | 3.76 | 45.8 | 311.27 | 85.62 | 8.89 |
|  | **Site 3** | **NS3L7** | 2.91 | 78.95 | 560.09 | 179.15 | 13.3 |
|  |  | **NS3L8** | 3.54 | 77.19 | 348.58 | 184.65 | 12.42 |
|  |  | **NS3L9** | 3.58 | 63.25 | 540.04 | 137.27 | 14.63 |
|  | **Mean ± SD** | | 3.40 ± 1.91 | 65.04 ± 22.86 | 454.57 ± 137.52 | 159.48 ± 52.97 | 14.51 ± 4.32 |
| **Baseline Value [81]** | | | 0.5 | 50 | N/A | 200 | 20 |
| **Reference Value in Residential Zone [81]** | | | 2 | 150 | N/A | 500 | 50 |
| **Reference Value in** **Industrial** **Zone [81]** | | | 10 | 500 | N/A | 1500 | 300 |

*N/A = Not Available*
