# Supplementary material for: Source apportionment and non-carcinogenic health risk assessment of selected heavy metals in the Turag river area of Gazipur District, Bangladesh
Source: PLoS One. 2026 Nov 6;21(11):e0362351. doi: 10.1371/journal.pone.0362351 (PMC13742873; doi:10.1371/journal.pone.0362351)
Supplement: Supplementary file 5 [file pone.0362351.s005.docx]

**S5 Table.** Metal concentrations (mg/L) in water samples collected from both the industrial and non-industrial zones (baseline and reference values [81]).

| **Zones** | **Sampling Sites** | **Sampling Locations** | **Hg** | **Ni** | **Mn** | **Zn** | **Co** |
| --- | --- | --- | --- | --- | --- | --- | --- |
| **Industrial** | **Site 1** | **IS1L1** | 2.69×10^-3^ | 1.26×10^-2^ | 1.38×10^-1^ | 1.27×10^-1^ | 1.70×10^-3^ |
|  |  | **IS1L2** | 2.23×10^-3^ | 5.98×10^-3^ | 5.89×10^-2^ | 8.54×10^-2^ | 3.60×10^-4^ |
|  |  | **IS1L3** | 2.2×10^-3^ | 5.04×10^-3^ | 7.87×10^-2^ | 4.25×10^-2^ | 4.00×10^-4^ |
|  | **Site 2** | **IS2L4** | 1.73×10^-3^ | 8.61×10^-3^ | 1.49×10^-1^ | 1.56×10^-1^ | 8.80×10^-4^ |
|  |  | **IS2L5** | 1.27×10^-3^ | 6.39×10^-3^ | 8.36×10^-2^ | 6.02×10^-2^ | 5.30×10^-4^ |
|  |  | **IS2L6** | 1.47×10^-3^ | 5.58×10^-3^ | 7.47×10^-2^ | 5.93×10^-2^ | 6.40×10^-4^ |
|  | **Site 3** | **IS3L7** | 1.33×10^-3^ | 9.49×10^-3^ | 1.42 | 9.30×10^-2^ | 1.03×10^-3^ |
|  |  | **IS3L8** | 1.03×10^-3^ | 8.04×10^-3^ | 5.90×10^-3^ | 1.10×10^-1^ | 5.40×10^-4^ |
|  |  | **IS3L9** | 9.80×10^-4^ | 5.15×10^-3^ | 6.82×10^-2^ | 4.65×10^-2^ | 3.70×10^-4^ |
|  | **Mean ± SD** | | 1.63×10^-3^  ±  5.87×10^-4^ | 7.42×10^-3^  ±  2.51×10^-3^ | 2.96×10^-1^  ±  4.54×10^-1^ | 8.16×10^-2^  ±  4.68×10^-2^ | 7.11×10^-4^  ±  4.31×10^-4^ |
| **Non-industrial** | **Site 1** | **NS1L1** | 1.29×10^-3^ | 4.47×10^-3^ | 3.66×10^-2^ | 1.26×10^-1^ | 3.10×10^-4^ |
|  |  | **NS1L2** | 1.73×10^-3^ | 4.73×10^-3^ | 5.45×10^-2^ | 3.61×10^-2^ | 3.50×10^-4^ |
|  |  | **NS1L3** | 1.15×10^-3^ | 4.89×10^-3^ | 5.91×10^-2^ | 7.51×10^-2^ | 4.20×10^-4^ |
|  | **Site 2** | **NS2L4** | 1.51×10^-3^ | 5.73×10^-3^ | 6.26×10^-2^ | 1.18×10^-1^ | 6.00×10^-4^ |
|  |  | **NS2L5** | 1.44×10^-3^ | 4.76×10^-3^ | 4.83×10^-2^ | 2.15×10^-2^ | 3.50×10^-4^ |
|  |  | **NS2L6** | 3.02×10^-3^ | 7.46×10^-3^ | 5.65×10^-2^ | 5.87×10^-2^ | 5.40×10^-4^ |
|  | **Site 3** | **NS3L7** | 3.91×10^-3^ | 4.83×10^-3^ | 4.75×10^-2^ | 1.30×10^-1^ | 3.50×10^-4^ |
|  |  | **NS3L8** | 2.52×10^-3^ | 4.97×10^-3^ | 4.63×10^-2^ | 1.19×10^-1^ | 3.40×10^-4^ |
|  |  | **NS3L9** | 2.76×10^-3^ | 4.29×10^-3^ | 3.39×10^-2^ | 3.99×10^-2^ | 3.30×10^-4^ |
|  | **Mean ± SD** | | 2.14×10^-3^  ±  9.45×10^-4^ | 5.13×10^-3^  ±  9.68×10^-4^ | 4.95×10^-2^  ±  9.77×10^-3^ | 8.05×10^-2^  ±  4.33×10^-2^ | 4.00×10^-4^  ±  1.00×10^-4^ |
| **Baseline Value [81]** | | | 4×10^-4^ | 1.00×10^-2^ | N/A | 5.00×10^-2^ | 5×10^-3^ |
| **Reference Value [81]** | | | 2×10^-3^ | 2.00×10^-1^ | N/A | 5 | 3.00×10^-1^ |

*N/A = Not Available*
