# Supplementary material for: Source apportionment and non-carcinogenic health risk assessment of selected heavy metals in the Turag river area of Gazipur District, Bangladesh
Source: PLoS One. 2026 Nov 6;21(11):e0362351. doi: 10.1371/journal.pone.0362351 (PMC13742873; doi:10.1371/journal.pone.0362351)
Supplement: Supplementary file 6 [file pone.0362351.s006.docx]

**S6 Table.** Chronic daily intake (CDI) of heavy metal-contaminated soil from the industrial and non-industrial zones through ingestion, dermal absorption, and inhalation (mg/day).

| **Zones** | **Pathways** | **Hg** | | **Ni** | | **Mn** | | **Zn** | | **Co** | |
| --- | --- | --- | --- | --- | --- | --- | --- | --- | --- | --- | --- |
|  |  | **Adult** | **Child** | **Adult** | **Child** | **Adult** | **Child** | **Adult** | **Child** | **Adult** | **Child** |
| **Industrial** | ***CDI_ing_*** | 7.09×10^-6^ | 6.62×10^-5^ | 2.48×10^-4^ | 2.31×10^-3^ | 7.13×10^-4^ | 6.66×10^-3^ | 2.91×10^-4^ | 2.72×10^-3^ | 1.74×10-5 | 1.62×10^-4^ |
|  | ***CDI_dermal_*** | 2.83×10^-8^ | 1.06×10^-7^ | 9.90×10^-7^ | 3.70×10^-6^ | 2.85×10^-6^ | 1.07×10^-5^ | 1.16×10^-6^ | 4.35×10^-6^ | 6.93×10^-8^ | 2.59×10^-7^ |
|  | ***CDI_inh_*** | 1.04×10^-9^ | 4.87×10^-9^ | 3.65×10^-8^ | 1.70×10^-7^ | 1.05×10^-7^ | 4.90×10^-7^ | 4.28×10^-8^ | 1.99×10^-7^ | 2.55×10^-9^ | 1.19×10^-8^ |
|  | ***CDI_total_*** | 7.12×10^-6^ | 6.63×10^-5^ | 2.49×10^-4^ | 2.32×10^-3^ | 7.16×10^-4^ | 6.67×10^-3^ | 2.92×10^-4^ | 2.72×10^-3^ | 1.74×10^-5^ | 1.62×10^-4^ |
| **Non-industrial** | ***CDI_ing_*** | 4.65×10^-6^ | 4.34×10^-5^ | 8.91×10^-5^ | 8.31×10^-4^ | 6.23×10^-4^ | 5.81×10^-3^ | 2.18×10^-4^ | 2.04×10^-3^ | 1.99×10^-5^ | 1.85×10^-4^ |
|  | ***CDI_dermal_*** | 1.86×10^-8^ | 6.95×10^-8^ | 3.55×10^-7^ | 1.33×10^-6^ | 2.48×10^-6^ | 9.30×10^-6^ | 8.72×10^-7^ | 3.26×10^-6^ | 7.93×10^-8^ | 2.97×10^-7^ |
|  | ***CDI_inh_*** | 6.84×10^-10^ | 3.19×10^-9^ | 1.31×10^-8^ | 6.11×10^-8^ | 9.16×10^-8^ | 4.27×10^-7^ | 3.21×10^-8^ | 1.50×10^-7^ | 2.92×10^-9^ | 1.36×10^-8^ |
|  | ***CDI_total_*** | 4.67×10^-6^ | 4.35×10^-5^ | 8.95×10^-5^ | 8.33×10^-4^ | 6.25×10^-4^ | 5.82×10^-3^ | 2.19×10^-4^ | 2.04×10^-3^ | 1.99×10^-5^ | 1.86×10^-4^ |
