# Supplementary material for: Source apportionment and non-carcinogenic health risk assessment of selected heavy metals in the Turag river area of Gazipur District, Bangladesh
Source: PLoS One. 2026 Nov 6;21(11):e0362351. doi: 10.1371/journal.pone.0362351 (PMC13742873; doi:10.1371/journal.pone.0362351)
Supplement: Supplementary file 7 [file pone.0362351.s007.docx]

**S7 Table.** Chronic daily intake (CDI) of heavy metal-contaminated water from the industrial and non-industrial zones through ingestion, dermal absorption, and inhalation (mg/day).

| **Zones** | **Pathways** | **Hg** | | **Ni** | | **Mn** | | **Zn** | | **Co** | |
| --- | --- | --- | --- | --- | --- | --- | --- | --- | --- | --- | --- |
|  |  | **Adult** | **Child** | **Adult** | **Child** | **Adult** | **Child** | **Adult** | **Child** | **Adult** | **Child** |
| **Industrial** | ***CDI_ing_*** | 4.99×10^-11^ | 1.91×10^-10^ | 2.24×10^-10^ | 8.55×10^-10^ | 8.92×10^-9^ | 3.40×10^-8^ | 2.61×10^-9^ | 9.97×10^-9^ | 2.16×10^-11^ | 8.25×10^-11^ |
|  | ***CDI_dermal_*** | 2.72×10^-10^ | 1.02×10^-9^ | 1.22×10^-9^ | 4.56×10^-9^ | 4.85×10^-8^ | 1.82×10^-7^ | 1.42×10^-8^ | 5.32×10^-8^ | 1.18×10^-10^ | 4.40×10^-10^ |
|  | ***CDI_total_*** | 3.22×10^-10^ | 1.21×10^-9^ | 1.44×10^-9^ | 5.42×10^-9^ | 5.74×10^-8^ | 2.16×10^-7^ | 1.68×10^-8^ | 6.31×10^-8^ | 1.39×10^-10^ | 5.22×10^-10^ |
| **Non-industrial** | ***CDI_ing_*** | 6.42×10^-11^ | 2.47×10^-10^ | 1.54×10^-10^ | 5.90×10^-10^ | 5.20×10^-12^ | 2.00×10^-11^ | 3.00×10^-10^ | 1.15×10^-9^ | 1.20×10^-11^ | 4.59×10^-11^ |
|  | ***CDI_dermal_*** | 3.52×10^-10^ | 1.32×10^-9^ | 8.40×10^-10^ | 3.15×10^-9^ | 1.49×10^-9^ | 5.69×10^-9^ | 8.11×10^-9^ | 3.04×10^-8^ | 6.54×10^-11^ | 2.45×10^-10^ |
|  | ***CDI_total_*** | 4.17×10^-10^ | 1.57×10^-9^ | 9.95×10^-10^ | 3.74×10^-9^ | 9.60×10^-9^ | 3.60×10^-8^ | 1.56×10^-8^ | 5.87×10^-8^ | 7.74×10^-11^ | 2.91×10^-10^ |
