# Supplementary material for: Source apportionment and non-carcinogenic health risk assessment of selected heavy metals in the Turag river area of Gazipur District, Bangladesh
Source: PLoS One. 2026 Nov 6;21(11):e0362351. doi: 10.1371/journal.pone.0362351 (PMC13742873; doi:10.1371/journal.pone.0362351)
Supplement: Supplementary file 8 [file pone.0362351.s008.docx]

**S8 Table.** Hazard quotient (HQ) and hazard index (HI) of heavy metal-contaminated water from both industrial and non-industrial zones.

| **Zones** | **Pathways** | **Adult** | | | | | **Child** | | | | |
| --- | --- | --- | --- | --- | --- | --- | --- | --- | --- | --- | --- |
|  |  | **Hg** | **Ni** | **Mn** | **Zn** | **Co** | **Hg** | **Ni** | **Mn** | **Zn** | **Co** |
| **Industrial** | ***HQ_ing_*** | 1.67×10^-8^ | 1.60×10^-7^ | 6.37×10^-8^ | 8.70×10^-9^ | 7.20×10^-8^ | 6.36×10^-8^ | 6.11×10^-7^ | 2.43×10^-7^ | 3.32×10^-8^ | 2.75×10^-7^ |
|  | ***HQ_dermal_*** | 1.30×10^-5^ | 6.77×10^-7^ | 8.66×10^-6^ | 1.89×10^-7^ | 2.35×10^-8^ | 4.85×10^-5^ | 2.53×10^-6^ | 3.24×10^-5^ | 7.09×10^-7^ | 8.80×10^-8^ |
|  | ***HQ_total_*** | 1.30×10^-5^ | 8.37×10^-7^ | 8.73×10^-6^ | 1.98×10^-7^ | 9.55×10^-8^ | 4.85×10^-5^ | 3.15×10^-6^ | 3.27×10^-5^ | 7.42×10^-7^ | 3.63×10^-7^ |
|  | ***HI*** | **Adult** | | | | | **Child** | | | | |
|  |  | 2.28×10^-5^ | | | | | 8.55×10-5 | | | | |
| **Non-industrial** | ***HQ_ing_*** | 2.16×10^-8^ | 1.10×10^-7^ | 1.06×10^-8^ | 8.09×10^-9^ | 4.01×10^-8^ | 8.24×10^-8^ | 4.21×10^-7^ | 4.07×10^-8^ | 3.09×10^-8^ | 1.53×10^-7^ |
|  | ***HQ_dermal_*** | 1.68×10^-5^ | 4.67×10^-7^ | 1.45×10^-6^ | 1.76×10^-7^ | 1.31×10^-8^ | 6.28×10^-5^ | 1.75×10^-6^ | 5.42×10^-6^ | 6.59×10^-7^ | 4.90×10^-8^ |
|  | ***HQ_total_*** | 1.68×10^-5^ | 5.77×10^-7^ | 1.46×10^-6^ | 1.84×10^-7^ | 5.32×10^-8^ | 6.28×10^-5^ | 2.17×10^-6^ | 5.46×10^-6^ | 6.90×10^-7^ | 2.02×10^-7^ |
|  | ***HI*** | **Adult** | | | | | **Child** | | | | |
|  |  | 1.91×10^-5^ | | | | | 7.14×10^-5^ | | | | |
